# Barriers and facilitators for weight management interventions in breast cancer patients: a systematic review of qualitative studies

**DOI:** 10.1080/17482631.2023.2259290

**Published:** 2023-10-15

**Authors:** Sheena Tjon A Joe, Sara Verschure-Dorsman, Erica A. Wilthagen, Martijn Stuiver

**Affiliations:** a clinical dietitian, The Netherlands Cancer Institute, Department of dietetics, Amsterdam, The Netherlands; b medical information specialist, Scientific Information Service, The Netherlands Cancer Institute, Amsterdam, The Netherlands; c Department of Epidemiology and Data Science, CCA, AmsterdamUMC, University of Amsterdam, Amsterdam, The Netherlands; d Associate group leader of the Cancer Survivorship group, Division of Psychosocial Research and Epidemiology, The Netherlands Cancer Institute, Amsterdam, The Netherlands

**Keywords:** Breast cancer, weight management, qualitative research, barriers and facilitators, lifestyle

## Abstract

**Introduction:**

This systematic review and meta-synthesis of qualitative studies provides an overview of barriers and facilitators that breast cancer patients experience in weight management interventions.

**Methods:**

We included qualitative studies describing barriers and facilitators for weight management interventions as experienced by adult breast cancer patients after the completion of initial treatment . The data was extracted and using thematic analysis.

**Results:**

After analysis, eleven themes were determined. Six of those themes could be linked to the Attitude, Social Influence and self Efficacy (ASE)-model. Physical and mental benefits, anticipated regret and a lack of motivation were linked to attitude. Integrating a weight management programme in daily life, stigma and fears were linked to self-efficacy. With regard to the social influence determinant, encouragement and discouragement by family members were developed as a theme. Four additional themes were conducted related to weight management behaviour; external barriers, economic barriers, cultural barriers and physical barriers. In addition, integrating weight management in cancer care was described as a separate theme.

**Conclusions:**

Several disease specific issues, including feeling stigmatized after cancer treatment and treatment-related side effects and peer-support should be given specific attention to maximize adherence of weight management programmes.

## Introduction

Unintended weight gain is one of the three most experienced long-term health problems among people treated for early-stage breast cancer and was first reported by J.K. Dixon et al. in 1978 (Dixon et al., 1978). Especially in those undergoing chemotherapy, bodyweight can increase significantly during treatment (van den Berg et al., 2017). A descriptive, correlational study found a significant increase of more than 2.5 kg in 63.5% of women one year after the start of treatment with chemotherapy, which negatively affected their quality of life (McInnes & Knobf, 2001). Even after two years, 68% of the women maintained a significant weight gain.

While the exact cause of unintended weight gain after breast cancer treatment remains unclear, current evidence suggests that treatment with chemotherapy results in more weight gain compared to localized treatment (surgery with or without radiation) alone (Demark-Wahnefried et al., 2001). Moreover, weight gain was more often seen in patients treated with more extensive protocols and multi-agent therapies (Vance et al., 2011). While an association with the use of tamoxifen alone has not been shown, corticosteroids like dexamethasone and prednisone, which are often prescribed during breast cancer treatment to treat nausea and inflammation, can increase appetite and can therefore contribute to structural weight gain (Faber-Langendoen, 1996; Goodwin et al., 1988).

Besides treatment-related side-effects, a younger age and treatment-induced premature menopause have been associated with excessive weight gain (Makari-Judson et al., 2014). Furthermore, it is plausible that behavioural change, like reduced physical activity, also contributes to unwanted weight gain. It is well documented that breast cancer survivors have reduced physical activity levels, compared to pretreatment, and compared to the general population (Broderick et al., 2014; Ee et al., 2020; Irwin et al., 2003).

Weight gain afte**r** initial treatment can eventually lead to overweight or obesity. Overweight and obese breast cancer survivors are at increased risk of cancer recurrence and have higher all-cause mortality (Anbari et al., 2019). Obesity also has a negative impact on breast cancer survivors’ quality of life (QoL), and it increases the risk of longer-term morbidities such as type 2 diabetes mellitus and cardiovascular disease (Anbari et al., 2019). Prevention of weight gain, both during treatment and in the survivorship phase, should therefore be given due consideration.

To date, there have been a limited number of weight management intervention studies, with short follow-up and small sample-sizes. Although the optimal weight loss intervention for breast cancer patients has not yet been determined, some studies on comprehensive multimodal weight loss interventions have shown promising effects on body weight, BMI, waist circumference and overall quality of life (Playdon et al., 2013; Shaikh et al., 2020).

For a weight management intervention to be successfully implemented, the barriers and facilitators for uptake of the intervention (components) should be clarified and adequately addressed in the intervention design.

Previous quantitative research has identified common barriers for healthy behaviour of breast cancer patients and survivors. This includes a high level of distress, fatigue, lack of motivation, psychosocial problems after breast cancer treatment, and a lack of service provision around weight gain prevention and weight management (Broderick et al., 2014; Ee et al., 2020; Howard-Anderson et al., 2012; Ventura et al., 2013).

Qualitative research enhances this knowledge, by providing in depth insights in patients’ personal motives, their views about essential components of weight management interventions, and the barriers and facilitators they experience (Evans, 2002). Qualitative meta-synthesis can indicate the level of overall saturation of these topics, and provide a structured summary of the available evidence, and thereby improve the interpretation of qualitative research findings (Goodman, 2008).

Therefore, the aim of this study is to provide a thematic overview of high-quality qualitative research investigating the barriers and facilitators that people with breast cancer experience for participating in and adhering to interventions regarding weight management. The results can be used in the further development and implementation of patient-oriented weight management interventions that are likely to be acceptable in clinical practice.

## Methods

A systematic review of the literature was conducted between July 2021 and April 2022. The protocol for the review was registered in PROSPERO under the ID number: CRD42021233420 on 22 April 2021: https://www.crd.york.ac.uk/PROSPERO. The PRISMA 2009 checklist was used as a guideline for reporting (Page et al., 2021).

### Database search

Four databases (Medline, Embase, Psychinfo, and Cinahl) were systematically searched for relevant qualitative papers, considering barriers and facilitators for weight management among breast cancer survivors. The search was conducted by an experienced medical information specialist. A forward and backward citation search was made, to avoid missing relevant papers. The complete search strategy is included in supplementary Appendix 1.

### Study selection

Studies were considered eligible for inclusion if they comprised qualitative research, such as focus group studies, semi-structured interviews, or mixed-methods studies. Eligible studies explored barriers and facilitators for exercise, diet and/or weight management programmes, in samples of adults (>18 yr), who had been diagnosed with breast cancer and who were not currently undergoing “active” treatment (defined as surgery, chemotherapy, radiotherapy). Studies among cancer survivors undergoing hormonal therapy were also eligible.

We excluded non-English studies. Furthermore, articles were also excluded when they did not describe original research (e.g., study protocols, synopses, or systematic reviews) or when full texts were not available (e.g., in case of congress abstracts).

To gain a broad perspective on barriers and facilitators for weight management programmes, we included studies describing outcome expectations of weight management programmes under development, as well as studies evaluating existing programmes (perceived barriers and facilitators).

Two researchers (ST and SVD) independently screened and labelled all retrieved records based on title and abstract using Rayyan QCRI software (Ouzzani et al., 2016). Disagreements were resolved by discussion. Of the remaining papers, full-text articles were retrieved for critical appraisal.

### Data extraction and quality assessment

Data on study design (data collection methods), sample characteristics (sample size, age, sex, cancer stage, and time since diagnosis) and participant selection details were extracted and described separately for each study (Table I). All text labelled as “results”, or “findings” were extracted electronically and entered in a qualitative data analysis computer programme (NVivo 10). Results included author narrative, as well as participant quotes. Data extraction forms were checked by the two reviewers separately to ensure accuracy.

**Table I.** Study characteristics.

First author, year	Title	Study type	Sample size/age	Diagnosis, treatment and time since treatment	Intervention + duration	Identified barriers	Identified facilitators
Balneaves L, 2014	Breast cancer survivors’ perspective on a weight loss and physical activity lifestyle intervention	Focus group or interviews by telephone	N=14Mean age 55.6 (SD 9.5) Mean age 55.6 (SD 9.5)	Stage II (67%) Stage III (11%) Unknown (22%) Surgery + radiation (22%) Surgery and chemo (11%) Surgery, chemo and radiation (67%) Time since treatment unknown	24 weeks lifestyle intervention	-Logistics of attending an intervention that required attendance two to three times a week -Fatigue -Negative reaction from their social network	-Inspired by the presence of other cancer survivors -Sharing experiences with like-minded peers
Balneaves L, 2020	Patient and Medical Oncologists’ Perspectives on Prescribed Lifestyle Intervention-Experiences of Women with Breast Cancer and Providers	One-on-one semi-structured interviews	N=12Mean age 51.9 (SD 14.2) Mean age 51.9 (SD 14.2)	Stage I (33%), Stage II (67%) Chemotherapy Time since treatment unknown	35 weeks lifestyle intervention, with exercise component of the NExT parent study	-Restricted gym hours (9 am-3 pm) -Location of the gym due to travel required -Life responsibilities such as childcare and employment	-Managing distress -Gaining social support -Coping with the physical consequences of cancer and beyond
Brunet J, 2015	Fostering positive experience of group-based exercise classes after breast cancer: what do women have to say?	Face to face Interviews	N=7Age range (40–69) Age range (40–69)	Breast cancer stage unknown Chemotherapy, radiation and hormone therapy (4) Hormone therapy (1) Chemo and radiation therapy (2) After completion of treatment	8 weeks exercise programme	-None mentioned	-Access to a qualified, supportive and motivated instructor -Personalized instruction focused on self-improvement
Brunet J, 2013	A qualitative exploration of barriers and motivators to physical activity participation in women treated for breast cancer	Semi-structured interviews	N=9Mean age 55.33 Mean age 55.33	Stage I-III Surgery (100%) Chemotherapy 73% Radiotherapy (82%) Hormone therapy 83%) Completed treatment <5 years (78%)	Physical activity	-Physical symptoms (cancer-related) -Environmental factors (bad weather, lack of equipment) -Psychosocial factors (social support, lack of motivation)	-Physical benefits (weight management, health benefits, increase energy) -Psychosocial benefits (improve body image, enjoyment, social support, positive emotions)
Bulmer S, 2012	Women's perceived benefits of exercise during and after breast cancer treatment	Interviews	N=45Mean age 53.1 (SD 6.24) Mean age 53.1 (SD 6.24)	Not mentioned	Exercise programme with 6 months unlimited access to health club facilities and personal training	-None mentioned	-Social benefits -Reclaiming their body -Feeling better -Health status -Rehabilitation -Managing emotions -Moving forward -Respite from cancer -Social benefits
Fazzino T, 2016	A qualitative evaluation of a group phone-based weight loss intervention for rural breast cancer survivors: Themes and mechanisms of success	Interviews	N=186Mean age 58.8 (SD 8.2) Mean age 58.8 (SD 8.2)	Stage 0: 8% Stage I: 41.4% Stage II: 34.5% Stage III: 16.1% Surgery (100%) Radiation (69,5%) Chemotherapy (67.2%) Hormone therapy (73.6%) Time since treatment 3.3 years (SD 2.4)	Weight loss intervention with structured meal plan and physical activity	-Daily physical activity -Daily self-monitoring -Food diary was time consuming	-Accountability to the group and to self -Convenience of the diet
Hefferon K, 2013	Understanding barriers to exercise implementation 5-year post-breast cancer diagnosis: a large-scale qualitative study	Semi-structured interviews	N=83Age range (29–76) Age range (29–76)	Stage 0-III Chemotherapy Radiation	Exercise	-Lack of motivation -Fears -Dislike of gym -Fatigue -Weight gain -Ageing process and co-morbidities -Employment -Tradition female care-giving activities -Proximity and/or access to facilities -Seasonal weather	-None mentioned
Hirschey R, 2017	Exploration of exercise outcome expectations among breast cancer survivors	Semi-structured interviews	N=20Mean age 62 (SD 8.5) Mean age 62 (SD 8.5)	Stage of disease not mentioned Surgery (100%), chemotherapy and radiation (70%) Time since treatment 4.2 year(2.3)	16 weeks aerobic exercise programme or a moderate intensity programme	-Fatigue	-Motivated to exercise to reduce the fear of recurrence
Husebo A, 2014	Factors perceived to influence exercise adherence in women with breast cancer participating in an exercise programme during adjuvant chemotherapy: a focus group study	Focus groups	N=27Mean age 52 (SD 9) Mean age 52 (SD 9)	Early stage breast cancer Chemotherapy and surgery Time since treatment unknown	Home based-exercise with resistance and aerobic training during chemotherapy, duration not mentioned	-Side effect of breast cancer treatment -Balancing time to exercise and social events	-Positive beliefs about efficacy and outcomes motivate exercise -Constructive support enhances exercise -Restoring and maintaining normality in daily life motivates exercise
Ingram C, 2010	Women's perceptions of home-based exercise performed during adjuvant chemotherapy for breast cancer	Interviews (open-ended questions)	N=8Mean age 47.5 (SD 10) Mean age 47.5 (SD 10)	Stage I-IIIA Surgery and chemotherapy (100%)	Exercise intervention with stretching, aerobic and strengthening 4 times/week, during chemotherapy 24 weeks	-Side effects of chemotherapy -Side effects of medication -Fatigue -Pain -Decreased range of motion (arm/shoulder) -Non-medical life events (vacation, caring for an ill relative, death of a friend)	-Internal motivation -External support -Adapting the routine
Jones L, 2020	Using the integrative model of behavioural prediction to understand female breast cancer survivors’ barriers and facilitators for adherence to a community-based group-exercise programme	Focus groups	N=17	Stage 0-III	6–12 months individualized exercise programme	-None mentioned	-Enjoyment -Exercising with other BC survivors -Comfortable atmosphere -One-on-one supervision -Physical changes -Psychological outcomes
Kim S, 2020	The experience of cancer-related fatigue, exercise and exercise adherence among women breast cancer survivors: insights from focus group interviews	Focus groups	N=16Mean age 48.4 (SD 8.4) Mean age 48.4 (SD 8.4)	Stage I (37.5%) Stage II (62.5%) Surgery and chemotherapy (100%) Radiation (75%) and hormone therapy (37.5%) After completion of surgery and chemotherapy, during radiation and/or hormone therapy	General exercise for an unknown period of time	-Fatigue	-Finding comfort and strength through exercising and interacting with other breast cancer survivors
Kokts-Porietis R, 2018	Breast cancer survivors’ perspectives on a home-based physical activity intervention utilizing wearable technology	Semi-structured interviews	N=6Mean age 58 (SD 7) Mean age 58 (SD 7)	Stage I (33.3%) Stage II (33.3%) Stage III (33.3%) Surgery, chemotherapy and radiation (67%) Hormone therapy (50%) Radiation therapy (66%) After completion of treatment, except for hormone treatment	12 weeks home-based physical activity intervention utilizing wearable technology	-Feeling busy -Lack of motivation -Weather	-Peer support -Progress in results
De Kruif A, 2020	Exploring changes in dietary intake, physical activity and body weight during chemotherapy in women with breast cancer: A Mixed-Methods study	In-depth semi-structured Interviews	N=25Mean age 5.4 range (25–67) Mean age 50.4 range (25–67)	Surgery and chemotherapy (100%)	Physical activity, dietary intake 150 min week physical activity, food diary	-Never liked to be active -Too tired -Side-effect from chemo -Changed appearance (hair loss, deterioration of breasts)	-Valued being active -Keeping fit -Contact with fellow patients
Leddy S, 1997	Incentives and barriers to exercise in women with a history of breast cancer	Semi-structured interviews	N=11Mean age 43 (SD 5.2) Mean age 43 (SD 5.2)	Chemotherapy (*N*=1) Hormone therapy (*N*=7) <3 years post treatment (*N*=9)	Exercise	-Lack of time -Not in routine -No partner -Dislike -Afraid -Hard -Expensive	-Benefit -Responsibility -Enjoyment -Previous experience -Family -Professionals -Fear of complications -Guilt
Lloyd G, 2020	Breast cancer survivors’ preferences for social support features in technology-supported physical activity interventions: finding from a mixed methods evaluation	Semi-structured interviews	N=28Mean age 53.4 (SD 1.1) Mean age 53.4 (SD 10.1)	Stage I (67.9%) Stage II (21.4%) Stage III (10.7%) Surgery (100%) Chemotherapy 50%) Radiation therapy (57%) Hormone therapy (57%) Months since treatment 21.8 (SD 15.1)	Technology supported physical activity intervention for an unknown period of time	-None mentioned	-Technology increased social connectedness -connecting with similar survivors
Loh S, 2010	Physical activity and women with breast cancer: insights from expert patients	Focus groups	N=14Mean age 55 (SD 8.7) Mean age 55 (SD 8.7)	Stage 0 (*n*=1) Stage I (*n*=4) Stage II (*n*=6) Stage III (*n*=2) Unknown (*n*=1) After treatment (*n*=6) During treatment (*n*=8) Treatment unknown Time since treatment unknown	General physical activity for unknown period of time	-Physical limitations -Weather -Age -Facilities -Role responsibility home/office commitment	-Positive experience from physical activity engagement -Easy access to facility engagement -Good social support
Mackenzie C, 2015	Breast cancer survivors’ experiences of partner support and physical activity participation	Semi-structured interviews	N=36Age range (28–52) Age range (28–52)	Time since diagnosis (11 months-20 years)	Exercise activity	-Fatigue -Domestic work -Childcare	-Supportive partners
Milosevic, 2020	Exploring tensions within young breast cancer survivors’ physical activity, nutrition and weight management beliefs and practices	Semi-structured interviews	N=12Mean age 36 (SD 3.4) Mean age 36 (SD 3.4)	Stage I-III Chemotherapy Within 5 years of diagnosis	General physical activity, nutrition and weight management during an unknown period of time	- Fatigue -Work and family responsibilities	-Exercise helped alleviate the stress -Physical activity helped to regain confidence in physical appearance
Monteiro-Guerra F, 2020	Breast cancer survivors’ perspectives on motivational and personalization strategies in mobile-app-based physical activity coaching interventions: qualitative study	Semi-structured Interviews	N=14Mean age 53.3 9SD 8.7) Mean age 53.3 9SD 8.7)	Stage I-III Surgery Chemotherapy Radiation therapy Time since diagnosis mean 4.25 (SD 2.8)	Exercise programme	-Lack of time -Physical limitations -Emotional challenges -Lack of information	-Social connectedness -Peer support -Confidence and perceived growth
Nock N, 2015	A community-based exercise and support group programme improves quality of life in African-American breast cancer survivors: A quantitative and qualitative analysis	Semi-structured phone interviews	N=19Mean age 56.5 (SD 11.0) Mean age 56.5 (SD 11.0)	Stage I (27.8%) Stage II (61.1%) Stage III (11.1%) Surgery, chemotherapy or irradiation <12 months after completion of treatment	20 weeks exercise and support intervention	-None mentioned	-Improvements in body composition, strength and function -Having fun
Nielsen A, 2019	Preferences for mHealth physical activity interventions during chemotherapy for breast cancer: a qualitative evaluation	Semi-structured phone interviews	N=30Mean age 45.5 (SD 9.6) Mean age 45.5 (SD 9.6)	Stage I (24.1%) Stage II (44.8%) Stage III (31.0%) Time since chemotherapy 5.7 months (SD 3.8) Time since radiation 59.3% Currently radiation 10% Currently hormone therapy 53.3%	Physical activity, goal: 150 min/week moderate-vigorous physical activity	-Side effects of treatment including nausea, fatigue, pain	-Education about PA during chemotherapy -Home-based programme -Personalized intervention -Social support from other breast cancer survivors, friends, family
Owusu C, 2018	Perspective of older African-American and non-Hispanic white breast cancer survivors from diverse socioeconomic backgrounds towards physical activity: A qualitative study	Interviews and focus groups	N=60Mean age 71 (65–87) Mean age 71 (65–87)	Stage I (53%) Stage II (33%) Stage III (24%) Surgery (100%) Radiation therapy (68%) Chemotherapy 30%) Hormone therapy (80%) <2 years of treatment completion	Physical activity	-Health issues -Side effects -Fatigue -Inclement weather -Lack of a PA buddy	-Increasing energy -Helping the body -Reducing stress -Helping emotionally and mentally -Religious faith -Encouraging family -Community environment
Piacentine L, 2018	Promoting team-based exercise among African American breast cancer survivors	Focus groups	N=12	Stage 0 (8%) Stage I (42%) Stage II (33%) Stage III (17%) Surgery (*n*=12) Chemotherapy (*n*=7) Radiotherapy (*n*=9)	14 week exercise and nutrition intervention	-Not wanting to exercise alone -Exercise is not a life or treatment priority -Side effects of cancer treatment (physical and emotional) -Fatigue	-Support by others -Less chance of recurrence -Mental benefits -General health, sleep, weight control -In the same boat -Improved activity
Pila E, 2018	‘The weight is even worse than the cancer’: Exploring preoccupation in women treated for breast cancer	Semi-structured interviews	N=84Mean age 66.3 (SD 1.2) Mean age 66.3 (SD 10.2)	Stage I (72.7%) Stage II (27.3%) Completed primary treatment 3.98 months (SD 3.07)	Weight management behavior	-Lack of willpower -Precipitating negative emotions -Self-criticism -Perceptions of failure	-Reducing breast cancer recurrence -Reducing comorbidities - Better appearance -Fitting societal ideas of how the body and weight should be
Power J, 2020	Experiences of African American breast cancer survivors using digital scales and activity trackers in a weight gain prevention intervention: Qualitative study	Semi-structured interviews	N=21Mean age 62.6 (SD 8.3) Mean age 62.6 (SD 8.3)	Completed primary treatment Years since diagnosis mean 3.38 (SD 2.46)	Weight gain prevention intervention; 6 months diet + physical activity	-Creation of routine (self-weighing) -Fluctuations in weight and increased awareness (self-weighing) -Forgetting or losing Daily Activity Tracking Device	-Improving QoL -Decrease risk of recurrence
Pullen T, 2018	Utilizing RE-AIM to examine the translational potential of Project MOVE, a novel intervention for increasing physical activity levels in breast cancer survivors	Focus groups, interviews	N=71Mean age 59.0 (SD 8.8)Years since diagnosis 9 years (SD 8.2) Mean age 59.0 (SD 8.8) Years since diagnosis 9 years (SD 8.2)	Stage 0 (6.9%) Stage I (14.9%) Stage II (24.1%) Stage III (14.9%) Stage IV (8.0%) Surgery (100%) Radiation therapy (54%) Chemotherapy (51.7%) Hormone therapy (28.7%)	Physical activity programme; Accelerometer, physical activity guidelines	-Pain -Fatigue -Treatment related side effects	-Small group size -Supportive environment -Health benefits -Group commitment − 500 dollar incentive
Van Puymbroeck M, 2013	Perceived health benefits from yoga among breast cancer survivors	Interview	N=18		Yoga intervention	-None mentioned	-Physical health & healing -Mental health & healing -Social health & healing
Rogers L, 2004	Exploring social cognitive theory constructs for promoting exercise among breast cancer patients	Focus groups	N=12Mean age 54 (SD 7.5) Mean age 54 (SD 7.5)	Non-metastatic BC Adjuvant treatment (17%) Months after treatment 16 months (SD 21)	Exercise	-Fatigue -Exercise not a priority -Obstacles of daily responsibilities -Nausea and malaise	-Improved survival -Reduced fatigue
Sander A, 2012	Factors that affect decisions about physical activity and exercise in survivors of breast cancer: A qualitative study	Focus groups	N=34Mean age 56.9 (SD 9.7) Mean age 56.9 (SD 9.7)	Stage 0 (*N*=1) Stage I (*N*=18) Stage II (*N*=8) Stage III (*N*=5) Surgery (*N*=43) Chemotherapy (*N*=19) Radiation therapy (*N*=30) Months after treatment 56.3 (SD 66.7)	Physical activity, exercise	-Fatigue -Neuropathy -Joint pain -Poor body image -Time -Musculoskeletal pain -Lymphedema	-Social support -Convenience
Shaw S, 2020	Cancer survivors’ experiences of an exercise programme during treatment and while employed: A qualitative pilot study	Semi-structured interviews	N=5Age range (51–58) Age range (51–58)	Stage IA-IIIA Chemotherapy and radiation therapy (100%)	Community-based exercise programme with one-on-one exercise therapy with resistance bands and free weight exercise; 2 sessions/week 40–45 min.	-none mentioned	-Mental well-being -Support from other BC-patients
Short C, 2013	How social cognitive theory can help oncology-based health professionals promote physical activity among breast cancer survivors	Semi-structured telephone interviews	N=8Mean age 55 range (44–63) Mean age 55 range (44–63)	Chemotherapy (100%) Radiation therapy (*N*=5) Hormone therapy (*N*=5) 4 years after treatment (range 3–6)	Exercise	-Lack of motivation -Health-issues -Changes in perspective after cancer -Not finding PA enjoyable	-Enjoyment -Increased energy -Feeling good mentally -Maintaining healthy weight -Sleep improvements -Sense of achievement -Preventing chronic disease -Fighting the ageing process
Smith S, 2015	Lifestyle modification experiences of African American breast cancer survivors: A needs assessment	Focus groups	N=42Mean age 45.73 (SD 7.91) Mean age 45.73 (SD 7.91)		Lifestyle modification needs	-‘pamphlet-only control group’	-peer-facilitated sessions -hands on or interactive nutrition education -support from co-survivors
Smith S, 2017	Community engagement to address socio-ecological barriers to physical activity among African American breast cancer survivors	Focus group	N=60Mean age 45.73 (7.91) Mean age 45.73 (7.91)		Physical activity programme; 24 weeks moderately intensive strength training and yoga	-Post-treatment symptoms (lymphoedema, neuropathy) -Fatigue -Body image -Co-morbidity -Perceptions -Lack of social support -Partner concerns -Lack of family support -Costs -PA preferences -Cultural issues -Lack of resources -Safety concerns -Weather	-None mentioned
Vassbakk-Brovold K, 2018	Experiences of patients with breast cancer of participating in a lifestyle intervention study while receiving adjuvant chemotherapy	Semi-structured interviews	N=10Mean age 53 range (27–68) Mean age 53 range (27–68)	Stage II & III Chemotherapy (100%)	Lifestyle intervention 12 months; diet, physical activity, stress management	-Adverse effects of chemotherapy -Feeling ‘overwhelmed’ -Lack of motivation -Prioritizing family and friends -Lack of energy -Reduced self-image -Tiredness	-Cooking recipes -Health benefits -More insight in own PA-level through counseling -Useful info about how to adjust PA to their health condition
Whitehead S, 2009	Older breast cancer survivors’ views and preferences for physical activity	Semi-structured interviews, focus group	N=29Mean age 66.54 (SD 6.5) Mean age 66.54 (SD 6.5)	1–5 years after diagnosis and treatment (except for hormone therapy)	Physical activity	-Lack of time -Health-related barriers, afraid of getting lymphoedema and soreness -Stiffness -Lack of motivation -Fatigue -Fear of ‘overdoing it’ post-illness -Self-consciousness	-Fight the ageing process -Control medical conditions -Weight loss -Self-image -Enjoyment -Be normal -Increased energy-levels -Well-being
Wu HS, 2019	Breast cancer survivors’ experiences with an activity tracker integrated into a supervised exercise programme: Qualitative study	Semi-structured in-depth interviews	N=10	12 months after diagnosis and treatment (except for hormone therapy)	Exercise programme with activity tracker, 12 weeks with supervised strength and endurance training 2x/week.	-Lack of personal advice -Not a realistic representation of total daily activity -No feedback from physiotherapist	-Activity tracker motivates -More awareness of lifestyle
Wurz A, 2015	Breast cancer survivors’ barriers and motives for participating in a group-based physical activity programme offered in the community	Interviews	N=7Mean age 55.3 Mean age 55.3	Surgery (100%) Chemotherapy/radiation therapy/hormone therapy (*N*=4) Radiation therapy/hormone therapy (*N*=2) Hormone therapy (*N*=1) Few months after treatment	Physical activity programme; 8 weeks group-based programme 2x/week	-Distance and traffic -Competing roles and responsibilities -Cancer-specific limitations	-Gaining social support -Networking -Being around similar others -Feeling a sense of personal fulfillment -Health benefits -Recovering from cancer
Yuve S, 2019	Lifestyle change experiences among breast cancer survivors participating in a pilot intervention: A narrative thematic analysis	Semi-structured interviews	N=4Age range (37–56) Age range (37–56)	Stage II & III Surgery, chemotherapy, radiation therapy (100%) Hormone therapy (*N*=2)	Healthy lifestyle; 12 weeks programme	-Perceived pressure to eat well and lose weight	-Scientific evidence to reduced cancer recurrence -Quality of life -Health benefits
Yufe S, 2021	Storying my lifestyle change: How breast cancer survivors experience and reflect on their participation in a pilot healthy lifestyle intervention	Semi-structured interviews	N=4	Stage I-III <5 years after chemotherapy and/or radiation therapy	Healthy lifestyle; 12 weeks programme	-Tiredness -Lack of motivation -Self-esteem	-Feel healthier

Quality appraisal was performed independently by two reviewers (ST and SVD) using the Critical Appraisal Skills Programme (CASP) (Zeng et al., 2015). The CASP is a widely used 10-item checklist, which systematically evaluates the internal validity, the external validity and the study results of each paper. Scoring of the items was done using reviewer guidelines adopted from Butler et al (Butler et al., 2016). Each reviewer assigned an overall quality rating of “high”, “medium”, or “low” to all papers. In case of disagreement, discussion was held until consensus was reached. Only high-quality studies were included in this systematic review.

### Theoretical framework and data synthesis

Analysing of the data was performed by a mixed-methods design. First, all extracted data was analysed using thematic synthesis as described by Thomas and Harden (Thomas & Harden, 2008). In the first stage, all fragments considering barriers, facilitators, preferences, experiences, essential components or requirements for a health intervention were extracted and coded line by line. In the second stage, two researchers generated and checked a preliminary code list. The moment that the most recent published articles didn’t contain anymore new codes, saturation was reached. Similar codes were collapsed, after which the codes were grouped into themes. In the third stage, themes were, if possible, linked to one of the core concepts of a theoretical framework. Multiple theoretical frameworks for behavioural change were considered and discussed by the researchers until consensus was reached. We used the social psychology model on behavioural intention (ASE-model), developed by de Vries et al. (see supplementary Appendix 2) (de Vries et al., 1995). In the ASE-model, it is assumed that behavioural intention and subsequent behaviour can be explained largely by three cognitive components; **A**ttitudes, **S**ubjective norm and self-**E**fficacy (ASE). A person’s attitude is formed by the cognitive and emotional consequences a person expects from this behaviour and the value attached to those consequences. Consequences include instrumental aspects (such as physical benefits) as well as emotional aspects (such as enjoyment or dislike). Subjective norm is the resultant of perceived social norms (i.e., due to experienced support or peer pressure), and the extent to which someone is inclined to conform to such norms. Self-efficacy refers to a person’s belief of his capability to perform and maintain the desired behaviour. In addition to the three main components of the ASE model, a person’s behaviour could also be influenced by environmental factors, such as the physical, cultural, or the economic environment (Congdon, 2019). These factors were therefore also considered as part of the theoretical framework. Remaining themes that did not fit the ASE-model were grouped into additional themes. Initial and final themes were discussed within the larger research group for triangulation.

## Ethics approval

As this study does not involve human participants, ethical approval does not apply.

## Results

**Figure 1.**
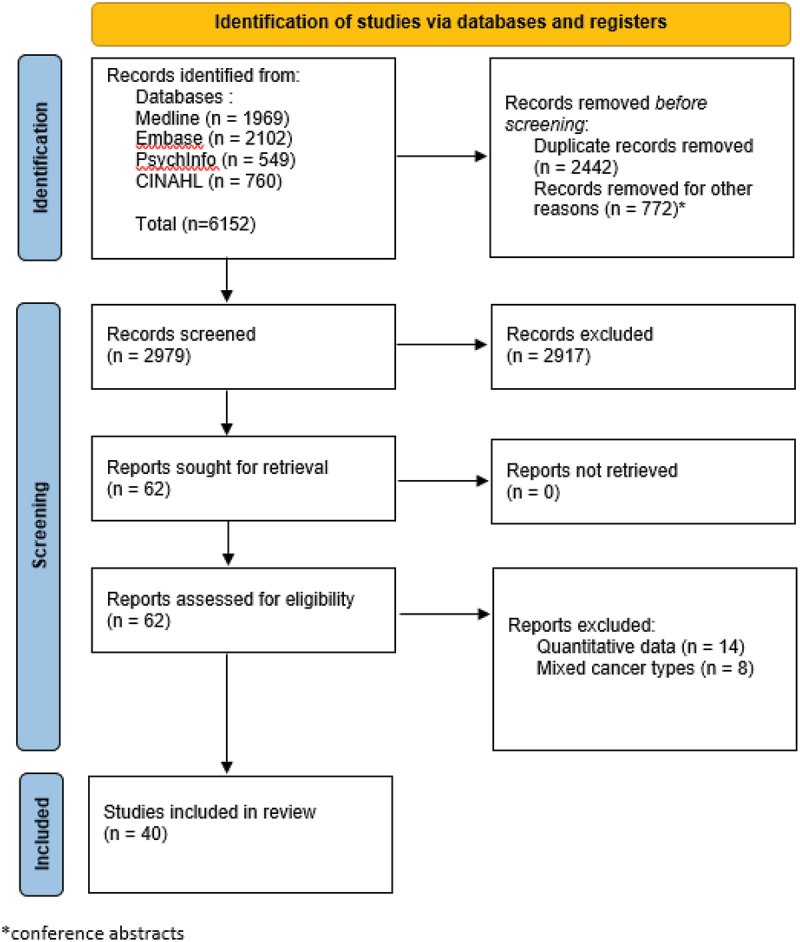
PRISMA 2020 flow diagram for new systematic reviews which included searches of databases and registers only.

A total of 6152 records were retrieved. After the removal of 2442 duplicates, 2979 titles and abstracts, and 62 full texts were screened for eligibility. Of the full texts, 40 studies were included after the initial search. A flow diagram of inclusion and exclusion of the retrieved publications is depicted in Figure 1.

Focus groups were used in nine studies, semi-structured interviews were used in 27 studies, and mixed-methods in four studies. Overall, one third of the studies was conducted in the USA, one third in Canada and one third in other countries. In 28 studies, an existing intervention was evaluated. The other 12 studies dealt with general views on nutritional and exercise programmes. Of all studies, 6 studies considered physical exercise and or nutrition (programmes) explicitly in the context of weight management, the remainder addressed physical activity or nutrition in a broader sense. For ease of reading, and since physical activity and nutrition are both essential elements in weight management, all interventions are further referred to as “weight management”, unless findings were specific for exercise or nutrition.

Disagreement in labelling occurred in less than 3% of the labels and was resolved through discussion in all cases.

Table I summarizes the included study characteristics and outcomes.

### Quality of the evidence

Although the relationship between de researcher and the participants of the study was not adequately considered in 85% of the articles, still all articles were considered high quality.

### Results of the data synthesis

After analysis, eleven themes were determined. Six of those themes could be linked to determinants in the ASE-model. Physical and mental benefits, anticipated regret and a lack of motivation were linked to *attitude*. Integrating a weight management programme in daily life, stigma and fears were linked to *self-efficacy*. The theme encouragement and discouragement by family members was linked to the *subjective norm* determinant. Besides the themes related to determinants of the ASE-model, four additional themes were developed related to weight management behaviour; external barriers, economic barriers, cultural barriers and physical barriers. In addition, integrating weight management in cancer care was described as a separate theme. Participant quotes are depicted in Table II to illustrate the developed themes.

**Table II.** Quotes and fragments illustrating the themes.

Theme	nodes	Exemplary quotes and fragments
Attitude	• Physical and mental benefits • Anticipated regret • Lack of motivation	The six most commonly reported expected outcomes or OEs included increased energy, feeling good overall, cardiac health, general health, feeling good mentally and achieving healthy weight (Nielsen et al., 2020). ..*a way to deal with the weight gain and body image issues that stemmed from treatment by addressing lifestyle factors that impact overall health, improving her QoL (Jones et al., 2020)*. *Overall, women described a strong and pervasive internalized pressure to maintain their weight, in both the context of reducing risk for cancer reoccurrence and dually linked to issues of appearance and fitting societal ideals of how the body and weight should be (Kokts-Porietis et al., 2019)*. *‘It helps me feel like I can clear my head’* (de Vries et al., 1995). *Some participants spoke about intrinsic rewards, such as* *feeling good after meeting the challenges they set for themselves, but more commonly participants spoke about negative reinforcers facilitating exercise, such as feeling guilty for not exercising (Vassbakk-Brovold et al., 2018)*. *Some participants suggested that their emotions were also affected after treatment and that it could negatively influence their motivation to participate in PA. Such emotional instability was reflected in feeling more stressed and depressed (Balneaves et al., 2014)*. *Other barriers relating to enrolment in the program were participants’ previous experiences with “gyms” and their attitudes toward the program. Preconceived ideas about the program varied among participants with some unsure, others concerned about enrollment due to it being a new experience or having had a prior negative experience with going to a “gym” (Balneaves et al., 2020)*.
Self-efficacy	• Integrating the programme in daily life • Stigma and fears	Work and “other commitments” were identified as being the most relevant and uncontrollable factors that adversely influenced level of adherence (Balneaves et al., 2020).Some hesitated joining activities in a group because of their hair loss and the deterioration of their breasts after surgery. They related their appearance to their female identity (Sander et al., 2012). *Some hesitated joining activities in a group because of their* *hair loss and the deterioration of their breasts after surgery. They related their appearance to their female identity (Sander et al., 2012)*.
Social influence		..a negative reaction from their social network. The women rationalized this response as representing fear on the part of family members and friends who confused weight loss with the progression of cancer (Owusu et al., 2018).
External barriers		I don’t want to get in the car and drive half an hour to do an activity. Something closer to home … somewhere around, but not too far … 10–15 maybe 20 min max, half an hour or an hour is too much’’ (de Vries et al., 1995).“The weather can be real depressing, and even if the weather kind of breaks, if I’ve been stuck inside, I just don’t want to do anything” (Whitehead & Lavelle, 2009) *“The weather can be real depressing, and even if the weather kind of breaks, if I’ve been stuck inside, I just don’t want to do anything” (Whitehead & Lavelle, 2009)*
Economical and cultural barriers		Prioritizing exercise over traditional female-caring roles can be a difficult decision for these Asian women with strong ties of extended family cultures, and many are living within an extended family system (Milosevic et al., 2020).
Physical barriers		The women expressed how a changed body image due to treatment affected their exercise adherence. A mastectomy required the use of a prosthesis, which had to be well adapted or else the women feared it would fall out during exercise. For most of the women, the chemotherapy also resulted in hair loss. They told that wearing a wig during exercise was not practical, and it also felt uncomfortable as soon as the physical activity (PA) made them start sweating (Kim et al., 2020).‘You can’t use your arm, almost forever. I enjoyed golf but now I had to quit, you know, because you’re not supposed to use your arm.’ (Piacentine et al., 2018) *‘You can’t use your arm, almost forever. I enjoyed golf but now I had to quit, you know, because you’re not supposed to use your arm.’ (Piacentine et al., 2018)*
Programme contents	• Timing • Setting • Execution	Oncologists and most patients believed the intervention should be offered as a standard part of breast cancer care, and as early as possible within the cancer trajectory, before the onset of treatment side effects could limit individuals’ enthusiasm and/or perceived capacity to participate (Pullen et al., 2019). *Survivors emphasized a desire for flexible coaching, suggesting a mix of scheduled calls, access to a coach during open office hours, and the ability to text questions anytime with answers returned within 24–48 hr (de Kruif et al., 2021)*. ..*their perceptions that yoga helped them physically heal while at the same time providing them with a venue for rediscovering and finding strength and confidence in their changed bodies (Wu et al., 2019)*. *Most survivors felt an exercise coach would be the most feasible in-person option and wanted this person to be highly trained in exercise and breast cancer treatment so they could provide accountability, encouragement and advice on setting goals, creating a workout plan, and overcoming barriers (Loh et al., 2011)*.

### Attitude

#### Physical and mental benefits

Besides weight control (Brunet et al., 2013; Leddy, 1997; Whitehead & Lavelle, 2009), physical benefits like improving general health, promoting recovery, increasing energy, improving sleep, and improving survival contributed to a positive attitude towards weight management interventions (Brunet et al., 2013; Mackenzie, 2015; Owusu et al., 2018; Rogers et al., 2004). These advantages were mostly experienced by participants who attended a weight management programme. Improved strength was mentioned in five papers (Balneaves et al., 2014; Bulmer et al., 2012; Monteiro-Guerra et al., 2020; Pullen et al., 2019; Wurz et al., 2015), more energy or feeling better overall was mentioned in as much as ten papers (Balneaves et al., 2014, 2020; Bulmer et al., 2012; Ingram et al., 2010; Jones et al., 2020; Monteiro-Guerra et al., 2020; Piacentine et al., 2018; Power et al., 2020; Pullen et al., 2019; Wurz et al., 2015). In four papers, participants described how the programme helped them manage their treatment-related side-effects (Balneaves et al., 2020; Bulmer et al., 2012; Kim et al., 2020; Wurz et al., 2015).

In three papers, it was stated that people derived enjoyment from exercise (Brunet et al., 2013; Jones et al., 2020; Nock et al., 2015), and in six papers, people described exercise or weight management as something positive they could do for themselves (Brunet et al., 2013; Bulmer et al., 2012; Husebø et al., 2015; Kokts-Porietis et al., 2019; Wurz et al., 2015; Yufe et al., 2019). Participants indicated that they expected benefit to psychological outcomes, like a better self-image and managing stress (Pila et al., 2018; Whitehead & Lavelle, 2009). This outcome expectation was met in many of the studies evaluating existing programmes for weight management, which found that participants felt mentally better and uplifted (Balneaves et al., 2020; Bulmer et al., 2012; Husebø et al., 2015; Ingram et al., 2010; Monteiro-Guerra et al., 2020; Nock et al., 2015; Owusu et al., 2018; Piacentine et al., 2018; Shaw et al., 2021; Wurz et al., 2015). In multiple papers, it was indicated that healthy lifestyle contributed positively to the transition from being a cancer patient to being a health-conscious individual (Balneaves et al., 2014, 2020; Bulmer et al., 2012; Husebø et al., 2015; Kokts-Porietis et al., 2019; Vassbakk-Brovold et al., 2018; Whitehead & Lavelle, 2009; Wurz et al., 2015).

#### Anticipated regret

Anticipated regret or moral obligation was indicated to be a motivator for a lifestyle programme. Moral obligation refers to the responsibility a person feels to, for instance, follow a lifestyle intervention because of personal beliefs and values. People commented that exercising was their own responsibility (Brunet et al., 2013; Fazzino et al., 2016; Leddy, 1997). Anticipated regret was mentioned in the sense that participants indicated that not following the programme gave them a bad conscience (Husebø et al., 2015; Short et al., 2013).

#### Lack of motivation

A negative affective attitude, on the other hand, in the sense of “not liking exercise”, or “not liking the gym” was mentioned in three different papers (Hefferon et al., 2013; Jones et al., 2020; Short et al., 2013). Some people experienced a lack of motivation for exercising in general or did not feel that exercising was a priority (Brunet et al., 2013; Rogers et al., 2004; Short et al., 2013; Whitehead & Lavelle, 2009). Even after evaluation of existing interventions, many people were not motivated, got bored with keeping up with the exercises, or missed the pleasure of good food (Fazzino et al., 2016; Hefferon et al., 2013; Husebø et al., 2015; Kokts-Porietis et al., 2019; Milosevic et al., 2020; Piacentine et al., 2018; Smith et al., 2017; Vassbakk-Brovold et al., 2018; Yufe et al., 2021). One person indicated that he was afraid that too much exercise could increase the risk of recurrence (Kim et al., 2020).

### Self-efficacy

#### Integrating the programme in daily life

Participants often struggled with combining a training programme or diet with work and/or household responsibilities. Conflicting priorities like going back to work, care giving tasks or social obligations were cited in 14 papers (Balneaves et al., 2014, 2020; Brunet et al., 2013; Hefferon et al., 2013; Husebø et al., 2015; Ingram et al., 2010; Mackenzie, 2015; Milosevic et al., 2020; Monteiro-Guerra et al., 2020; Piacentine et al., 2018; Sander et al., 2012; Whitehead & Lavelle, 2009; Wurz et al., 2015; Yufe et al., 2019). A lack of time and scheduling conflicts were often referred to as barriers for incorporating lifestyle changes in their daily life (Jones et al., 2020; Leddy, 1997; Loh et al., 2011; Milosevic et al., 2020; Rogers et al., 2004; Short et al., 2013; Whitehead & Lavelle, 2009; Wurz et al., 2015).

#### Stigma and fears

Also, on a psychological level, people experienced limitations that lowered their level of self-efficacy.

Participants described the impact of physical side-effects like hair-loss or a changed body after mastectomy on their self-esteem, and how this made them feel stigmatized and kept them from exercising in public (Brunet et al., 2013; de Kruif et al., 2021; Kim et al., 2020; Nielsen et al., 2020; Power et al., 2020; Sander et al., 2012; Smith et al., 2017; Whitehead & Lavelle, 2009; Yufe et al., 2021). Impaired concentration attributed to chemotherapy treatment was also mentioned (Balneaves et al., 2014). Participants were worried that exercise would exacerbate lymphoedema, or they associated exercise with physical pain (Rogers et al., 2004). Fear of exposure to infection was also mentioned in multiple papers (Loh et al., 2011; Nielsen et al., 2020; Rogers et al., 2004).

### Social influence

#### Encouragement and discouragement by family members and peers

Subjective norm played an important role in participant’s lifestyle habits. Support from family or close ones from a non-cancer environment was a strong motivator (Balneaves et al., 2014; Brunet et al., 2013; Bulmer et al., 2012; Husebø et al., 2015; Loh et al., 2011; Mackenzie, 2015; Monteiro-Guerra et al., 2020), as was the support of fellow cancer patients (Balneaves et al., 2014, 2020; Bulmer et al., 2012; de Kruif et al., 2021; Fazzino et al., 2016; Jones et al., 2020; Lloyd et al., 2020; Loh et al., 2011; Mackenzie, 2015; Milosevic et al., 2020; Nielsen et al., 2020; Nock et al., 2015; Piacentine et al., 2018; Pullen et al., 2019; Rogers et al., 2004; Shaw et al., 2021; Whitehead & Lavelle, 2009; Wurz et al., 2015). The need for an exercise buddy was mentioned in three papers (Brunet et al., 2013; Owusu et al., 2018; Piacentine et al., 2018).

In a few studies, participants mentioned being discouraged to exercise by their family due to the fear of being infected (Loh et al., 2011; Nielsen et al., 2020). Also, family members sometimes encouraged patients to rest (Sander et al., 2012), they misinterpreted weight loss as an indication of progression of cancer (Balneaves et al., 2014), or feared that high intensity of exercise might induce cancer recurrence (Kim et al., 2020).

### External barriers

Travel distance, poor access to or inadequacy of physical exercise facilities, restricted gym hours, or the absence of suitable equipment was found to be an external barrier for attending or maintaining exercise sessions in the context of weight management (Balneaves et al., 2014; Brunet et al., 2013; Hefferon et al., 2013; Piacentine et al., 2018; Short et al., 2013; Smith et al., 2017). Moreover, in some existing programmes, exercises were deemed too difficult, or not properly explained (Balneaves et al., 2020; Husebø et al., 2015; Kim et al., 2020; Piacentine et al., 2018). Sometimes, filling out questionnaires or writing up daily food intake was experienced as too time consuming (Fazzino et al., 2016; Vassbakk-Brovold et al., 2018). Weather conditions was mentioned as a barrier for exercising outdoors in 11 papers (Brunet et al., 2013; Fazzino et al., 2016; Hefferon et al., 2013; Ingram et al., 2010; Jones et al., 2020; Kokts-Porietis et al., 2019; Loh et al., 2011; Monteiro-Guerra et al., 2020; Owusu et al., 2018; Rogers et al., 2004; Smith et al., 2017).

### Economical and cultural barriers

In six studies, participants indicated that costs were a barrier for participating or maintaining a healthy lifestyle or participating in a a weight management programme (Brunet et al., 2013; Hefferon et al., 2013; Rogers et al., 2004; Short et al., 2013; Smith et al., 2017; Whitehead & Lavelle, 2009). In two papers, cultural barriers, in the sense of prioritizing a weight management programme over traditional female-caring roles was a problem (Loh et al., 2011; Smith et al., 2017). For instance, this was found in a study conducted in Malaysia, where participants were women with strong ties of extended family culture (Loh et al., 2011).

### Physical barriers

Participants experienced many physical impairments due to treatment-related side-effects, or comorbidities that kept them from exercising or from maintaining a weight management programme. Extreme fatigue, a lack of energy and complaints such as nausea or dizziness constituted a serious challenge, as recorded in 20 papers (Balneaves et al., 2014, 2020; Brunet et al., 2013; Fazzino et al., 2016; Hirschey et al., 2017; Husebø et al., 2015; Ingram et al., 2010; Jones et al., 2020; Kim et al., 2020; Loh et al., 2011; Mackenzie, 2015; Monteiro-Guerra et al., 2020; Nock et al., 2015; Power et al., 2020; Pullen et al., 2019; Rogers et al., 2004; Short et al., 2013; Vassbakk-Brovold et al., 2018; Wurz et al., 2015; Yufe et al., 2021). Lymphoedema was indicated as a barrier for exercise in ten papers (Hefferon et al., 2013; Kim et al., 2020; Milosevic et al., 2020; Monteiro-Guerra et al., 2020; Owusu et al., 2018; Pullen et al., 2019; Sander et al., 2012; Short et al., 2013; Whitehead & Lavelle, 2009; Wurz et al., 2015). Sometimes, unplanned hospital admissions or medical complications disrupted the programme (Husebø et al., 2015; Ingram et al., 2010). Moreover, women indicated that wearing breast prostheses or a wig were a barrier to attend classes (Husebø et al., 2015; Kim et al., 2020; Nock et al., 2015; Whitehead & Lavelle, 2009). In two papers, participants indicated that the weight gain itself was a barrier for exercise (Hefferon et al., 2013; Monteiro-Guerra et al., 2020).

### Integrating weight management in cancer care

In three papers, it was stated by the participants that a weight management intervention should be part of standard breast cancer care and offered early in the cancer treatment trajectory (Balneaves et al., 2014, 2020; Piacentine et al., 2018). A preference for home-based exercises, outside the hospital setting was also mentioned (Brunet et al., 2013; Husebø et al., 2015; Milosevic et al., 2020), although participants also indicated the need for support of knowledgeable professionals with extensive experience in guiding breast cancer patients (Balneaves et al., 2014; Lloyd et al., 2020; Nielsen et al., 2020; Nock et al., 2015; Rogers et al., 2004; Whitehead & Lavelle, 2009). Personalized and one-on-one supervision was frequently mentioned as important (Brunet & St-Aubin, 2016; Jones et al., 2020; Kim et al., 2020; Pullen et al., 2019; Wu et al., 2019). In one study, the need for specific information about safety and health benefits of exercise and weight maintenance during chemotherapy was discussed (Nielsen et al., 2020). A motivating and encouraging instructor was considered desirable (Brunet & St-Aubin, 2016; Bulmer et al., 2012; Husebø et al., 2015) and participants valued regular check-ups to monitor their performance (Ingram et al., 2010; Kokts-Porietis et al., 2019; Lloyd et al., 2020; Nielsen et al., 2020). With regard to supportive materials, menu planning and healthy recipes were considered convenient (Balneaves et al., 2014; Nock et al., 2015), as were easy-to-use individualized apps (Monteiro-Guerra et al., 2020; Smith et al., 2015). Finally, Yoga as a part of a lifestyle programme was considered of additional value by participants in three papers (Milosevic et al., 2020; Rogers et al., 2004; Van Puymbroeck et al., 2013).

## Discussion

With this systematic review and qualitative meta synthesis, we aimed to summarize the findings from the available qualitative literature, to inform the development and implementation of weight management interventions for people with breast cancer.

We found that barriers and facilitators for weight management interventions fit largely within the ASE model. That is: reported barriers are generally related to attitude, subjective norms and self-efficacy. Additionally, external barriers including economic, cultural, and physical barriers were identified. Some of these were cancer specific, such as fear of worsening symptoms or experiencing stigma. We also elicited views and preferences related to how to best integrate weight management into cancer care. Here, the importance of early attention to weight management and personalized support from knowledgeable professionals stood out.

### Implications for developing weight management interventions

The meta-synthesis has several implications for the development and implementation of weight management programmes for people with breast cancer. First, for effecting behavioural change, ensuring social support is essential. This could be achieved by engaging patients’ own support system or through patient support groups. In particular, attention should be given to sources of subjective norms that actively discourage physical activity behaviour. Successful results have indeed been shown in intervention studies where partners and family were involved in lifestyle programmes as well (Dorfman et al., 2022; George et al., 2020).

Second, physical side-effects from cancer or its treatment, such as extreme fatigue, loss of energy and (fear of) lymphoedema have consistently shown to be barriers for attending a weight management programme and need to be addressed. Our findings show that this is not only because of the symptom burden, but also because of possible negative outcome expectations with regard to the symptoms. It is therefore essential that healthcare professionals appoint these complaints and explain that a healthy lifestyle and exercise is not only safe, but also likely to reduce such side-effects (Juvet et al., 2017).

Timing and setting of a weight management intervention are essential to consider. In the studies included in this review, most patients would prefer to start during treatment or early in the trajectory. This is supported by intervention studies, which have shown promising results of the effect of exercise during, or right after treatment on side-effects and health related quality of life (Dieli-Conwright et al., 2018; Mishra et al., 2012). Patients generally preferred to be professionally monitored in a nearby facility. This suggests that although weight management programmes should be initiated in the hospital, they should preferably be offered—at least in part—in primary care or community settings.

For future weight management programmes, all of the above-mentioned factors should be included in a combined intervention, of which the feasibility and effectiveness have to be tested in a randomized controlled trial.

## Strengths and limitations

Notable strengths of this systematic review include the extensive systematic search supported by an experienced clinical librarian and the systematic quality appraisal.

To gain a broad perspective on the subject, this review included studies describing possible outcome expectations of a weight management programme or interventions that could be part of weight management, as well as studies evaluating existing programmes. The distinction between these categories was sometimes ambiguous. However, this difficulty has been addressed by getting two different authors agree on the thematic analysis and by using an explicit framework to organize the findings. A limitation of this is study is that non-English studies were excluded and consequently, most of the included studies were from the US or Canada which could lead to overemphasis on culturally dependent findings. However, corresponding themes were found in studies from European countries, Australia and New Zealand, which makes it plausible that most outcomes are universal.

Examining healthcare professionals’ perspectives was beyond the scope of our study, but could also provide new insights on possible barriers and facilitators in weight management with breast cancer patients. This would be useful to investigate in future research.

## Conclusion

In conclusion, breast cancer patient views and experiences about weight management programmes fit largely within the more generic behavioural framework of the ASE model. Within the concepts of the ASE model, several disease specific issues were identified including feeling stigmatized after cancer treatment and physical. Side effects like extreme fatigue and lymphoedema, and the motivating effect of social support from fellow survivors should be given specific attention.

## Appendix 1: Search strategy

### Medline (ovid)

**Table:**

#	Searches
1	exp breast neoplasms/OR ((breast* or mamma*) adj3 (neoplas* or tumor* or tumour* or cancer* or malign* or oncolog* or carcinom* or sarcoma* or lymphoma*)).ti,ab,kf.
2	exp health promotion/or (((weight or BMI or “body mass index” or “fat mass” or “fat percentage” or “fat distribution” or “muscle mass” or “body fat”) adj3 (maintain or maintaining or managing or manage* or maintenance)) or ((“weight loss” or weightloss or “weight reduct*” or “weight decreas*” or “weight gain” or diet or dietary or “healthy eating” or nutrition* or “eating behaviour” or lifestyle or “food intake” or exercise or “physical activit*” or health-coach*) adj5 (intervention* or programme* or recommendation* or support or promote or promoting or training*))).ti,ab,kf.
3	(barrier* or facilitat* or facilitating or motivat* or challenge* or obstacle* or adher* or enabler* or preference* or constraint* or “planned behav*”).ti,ab,kf. or (exp qualitative research/OR exp focus groups/or exp interview/or exp interviews as topic/or exp narration/or exp personal narratives as topic/or exp tape recording/or exp “surveys and questionnaires”/or ((qualitative adj3 (research* or analys* or stud* or method* or synthes* or approach*)) or “thematic analys*” or “content analys*” or “focus group*” or discussion* or interview* or narration* or narrative or “audio recording*” or audiotape* or “tape recording*” or survey* or questionnaire* or “patient report*” or “patient outcome*”).ti,ab,kf.)
4	1 and 2 and 3

### Embase.com

**Table:**

#	Searches
1	“breast cancer”/exp OR ((breast* OR mamma*) NEAR/3 (neoplas* OR tumor* OR tumour* OR cancer* OR malign* OR oncolog* OR carcinom* OR sarcoma* OR lymphoma*)):ti,ab,kw
2	“health promotion”/exp OR (((weight OR BMI OR “body mass index” OR “fat mass” OR “fat percentage” OR “fat distribution” OR “muscle mass” OR “body fat”) NEAR/3 (maintain OR maintaining OR managing OR manage* OR maintenance)) OR ((“weight loss” OR weightloss OR “weight reduct*” OR “weight decreas*” OR “weight gain” OR diet OR dietary OR “healthy eating” OR nutrition* OR “eating behaviour” OR lifestyle OR “food intake” OR exercise OR “physical activit*” OR health-coach*) NEAR/5 (intervention* OR programme* OR recommendation* OR support OR promote OR promoting OR training*))):ti,ab,kw
3	“programme acceptability”/exp OR (barrier* OR facilitat* OR facilitating OR motivat* OR challenge* OR obstacle* OR adher* OR enabler* OR preference* OR constraint* OR “planned behav*”):ti,ab,kw OR (“qualitative research”/exp OR “content analysis”/exp OR “thematic analysis”/exp OR “interview”/exp OR “narrative”/exp OR “recording”/exp OR “questionnaire”/exp OR ((qualitative NEAR/3 (research* OR analys* OR stud* OR method* OR synthes* OR approach*)) OR “thematic analys*” OR “content analys*” OR “focus group*” OR discussion* OR interview* OR narration* OR narrative OR “audio recording*” OR audiotape* OR “tape recording*” OR survey* OR questionnaire* OR “patient report*” OR “patient outcome*”):ti,ab,kw)
4	1 and 2 and 3

### PsycINFO

**Table:**

#	Searches
1	exp Breast Neoplasms/or ((breast* or mamma*) adj3 (neoplas* or tumor* or tumour* or cancer* or malign* or oncolog* or carcinom* or sarcoma* or lymphoma*)).ti,ab,id.
2	exp Health Promotion/or (((weight or BMI or “body mass index” or “fat mass” or “fat percentage” or “fat distribution” or “muscle mass” or “body fat”) adj3 (maintain or maintaining or managing or manage* or maintenance)) or ((“weight loss” or weightloss or “weight reduct*” or “weight decreas*” or “weight gain” or diet or dietary or “healthy eating” or nutrition* or “eating behaviour” or lifestyle or “food intake” or exercise or “physical activit*” or health-coach*) adj5 (intervention* or programme* or recommendation* or support or promote or promoting or training*))).ti,ab,id.
3	exp Programme Evaluation/or (barrier* or facilitat* or facilitating or motivat* or challenge* or obstacle* or adher* or enabler* or preference* or constraint* or “planned behav*”).ti,ab,id. or (exp qualitative methods/or exp surveys/or exp questionnaires/or ((qualitative adj3 (research* or analys* or stud* or method* or synthes* or approach*)) or “thematic analys*” or “content analys*” or “focus group*” or discussion* or interview* or narration* or narrative or “audio recording*” or audiotape* or “tape recording*” or survey* or questionnaire* or “patient report*” or “patient outcome*”).ti,ab,id.)
4	1 and 2 and 3
